# Misidentification of sex for *Lampsilis teres*, Yellow Sandshell, and its implications for mussel conservation and wildlife management

**DOI:** 10.1371/journal.pone.0197107

**Published:** 2018-05-16

**Authors:** Megan C. Hess, Kentaro Inoue, Eric T. Tsakiris, Michael Hart, Jennifer Morton, Jack Dudding, Clinton R. Robertson, Charles R. Randklev

**Affiliations:** 1 Texas A&M Natural Resources Institute, Texas A&M AgriLife Research Center at Dallas, Dallas, Texas, United States of America; 2 Texas Parks and Wildlife, Inland Fisheries Division, San Marcos, Texas, United States of America; Duke University Marine Laboratory, UNITED STATES

## Abstract

Correct identification of sex is an important component of wildlife management because changes in sex ratios can affect population viability. Identification of sex often relies on external morphology, which can be biased by intermediate or nondistinctive morphotypes and observer experience. For unionid mussels, research has demonstrated that species misidentification is common but less attention has been given to the reliability of sex identification. To evaluate whether this is an issue, we surveyed 117 researchers on their ability to correctly identify sex of *Lampsilis teres* (Yellow Sandshell), a wide ranging, sexually dimorphic species. Personal background information of each observer was analyzed to identify factors that may contribute to misidentification of sex. We found that median misidentification rates were ~20% across males and females and that observers falsely identified the number of female specimens more often (~23%) than males (~10%). Misidentification rates were partially explained by geographic region of prior mussel experience and where observers learned how to identify mussels, but there remained substantial variation among observers after controlling for these factors. We also used three morphometric methods (traditional, geometric, and Fourier) to investigate whether sex could be more correctly identified statistically and found that misidentification rates for the geometric and Fourier methods (which characterize shape) were less than 5% (on average 7% and 2% for females and males, respectively). Our results show that misidentification of sex is likely common for mussels if based solely on external morphology, which raises general questions, regardless of taxonomic group, about its reliability for conservation efforts.

## Introduction

Correct identification of sex is an important component of wildlife management for both game and non-game species. Ideally, all studies should hold concern with errors in estimation of sex, but the results of misidentification on conservation efforts focused on imperiled species raise the stakes considerably. In general, changes in sex ratio influence population growth as well as increase or decrease the risk of genetic problems [1,2], therefore sex ratios, and similar demographic parameters like age data, are useful for characterizing population viability. Identification of sex for most wildlife species typically relies on external morphology of live individuals or physical examination of the remains of the animal [2–4]. However, correct identification can be hindered by intermediate or nondistinctive morphotypes as well as observer-level factors, such as experience [2–4]. The consequences of misidentification of sex are unknown but presumably for game species can result in too restrictive or too liberal harvest regulations. For rare, non-game, taxa it can likely lead to generalizations about viability or misinterpretation of human related impacts and as a consequence confound conservation and management efforts [1–5].

Unionid mussels (hereafter, mussels) are considered one of the most imperiled groups of aquatic organisms in North America [6,7]. This is due to many factors including sensitivity to human impacts coupled with their immobility and unique life history, which relies on a host (usually a fish) to complete their reproductive life cycle [5]. The loss of mussels will likely have long-term ecological consequences for freshwater ecosystems because they influence nutrient cycling [8,9], enhance sediment stability [10], provide physical habitat for other benthic macroinvertebrates [11] and are forage for fish, birds, small mammals, and turtles [5]. To address this crisis, managers and mussel conservationists are beginning to develop risk assessment models that link extinction risk with demographic and genetic changes and use demographic-age information to evaluate different adaptive management strategies [12,13].

For mussels, sex ratios can be equal (e.g. [14]) or skewed toward one sex (e.g., [15,16]) and the ecological significance of this is poorly understood [5]. It is thought that in some cases the departure from a 1:1 sex ratio stems from biases related to sample size and methodology [5]. However, misidentification of sex is also a possible reason for skewed estimates of sex ratios. Methods for assessing sex of mussels vary depending on objectives but can include: 1) using shell shape of sexually dimorphic species [17–19]; 2) visually inspecting gills to assess gravidity [20–23]; 3) extracting gonadal fluid [24–26]; and 4) histological methods [14,23,27–30]. Of these approaches, shell shape is the easiest method to use to sex mussels because it can be done in the field, does not require vouchering, and costs very little in terms of time and effort. However, surveyors using shell shape to determine sex do so under the assumption that it’s done with minimum error, which is unlikely given recent research findings on species misidentification rates based on external morphology [31]. Morphological characteristics, which include shell shape, can vary based on habitat [32], stream position [33], and age of individuals [34], and these can exacerbate species misidentifications. Thus, for sexually dimorphic species it is safe to presume that these same factors could result in morphotypes that are likely to confound correct identification of sex.

Given the potential conservation issues related to misidentification of sex and the fact that this problem may be pervasive and not well recognized, we evaluated misidentification rates for *Lampsilis teres*, Yellow Sandshell, a sexually dimorphic and widely distributed mussel throughout North America. The specific objectives of this study were to: 1) survey the accuracy of researchers at identifying sex; 2) assess if sex of *L*. *teres* could be identified more correctly using three morphometric methods (traditional, geometric, and Fourier); and 3) identify factors that contribute to and potential solutions for minimizing misidentification.

## Methods

### Sampling

*Lampsilis teres* is found throughout the Mississippi River and the Gulf of Mexico basins. Similar to other species within the Lampsilini tribe, females tend to have a truncated posterior end, whereas males usually have a pointed posterior end [17,35]. A total of 50 individuals of *L*. *teres* were collected from Yegua Creek (30.368459° -96.343651°), a small tributary of the Brazos River, Texas [36] and 55 individuals were collected from the East Fork of the Trinity River (32.599597° -96.484854°). The latter were used to survey whether morphological variation observed between males and females at Yegua Creek was characteristic of *L*. *teres*. Field collections were conducted on public property and no specific permissions were required and our study did not involve a federally threatened/endangered or state protected species. Within Texas, *L*. *teres* is considered stable and is currently not listed or under consideration for state listing.

### Initial sex identification

Sex was determined by extracting gonadal fluid from each collected individual by inserting a 20-gauge hypodermic needle through the foot, positioned midline to the shell and half way into the visceral mass. Approximately 0.25–0.50 ml of gonadal fluid per individual was collected, fixed in 10% buffered formalin, and placed on ice for transport to the laboratory. Sex was then identified for each sample by adding methylene blue and using optical microscopy to identify spermatozoa (males) or oocytes (females) [36].

### Shell specimen preparation

In the laboratory, specimens were separated into soft tissue and shells, and the shells were scrubbed inside and out to remove excess tissue. All specimens were measured by taking the maximum length (anterior to posterior), height (dorsal to ventral), and width (right to left valve) to the nearest 0.1 mm using digital calipers (iGaging OriginCal); this information was then used for the traditional morphometric analyses. In preparation for the geometric and Fourier analyses, the right valve of each specimen was placed on a sheet with radial lines extended every 5° in a circle, and digital photographs of the external view were taken with a Canon EOS7D SLR camera. The outline of the shell was then extracted by cropping the image using Adobe^®^ Photoshop^®^ CC software v2015.0.0 (Adobe Systems).

### Identification survey

To evaluate misidentification rates of sex, observers were solicited at the 2017 Annual Meeting of the Texas Chapter of the American Fisheries Society and the 10^th^ Biennial Symposium of the Freshwater Mollusk Conservation Society. These identification surveys were non-experimental and voluntary and were conducted for educational purposes by the Texas Parks Wildlife Department, university researchers then analyzed the resulting data. The overall objective of these surveys was to improve workshops and trainings for professionals on mussel identification. Participants gained greater knowledge of mussel species and improved their identification skills via subsequent discussions. The resulting data contained no personal identifiers and there were no tangible incentives and so these activities were exempted from IRB. Informed consent from participants was not obtained as the data was analyzed anonymously.

A total of 117 observers were surveyed across the two meetings. Observers were given a brief set of instructions, a survey sheet, and shown a photo example of sexual dimorphism in *L*. *teres*. TPWD staff did not formally train observers beyond showing the photo example and so presumably the results of the test represent an unbiased assessment of sex identification. The survey sheet included the following 8 sections to complete: 1) academic background; 2) employment; 3) identification frequency (hours per month); 4); identification frequency (days per year); 5) mussel training; 6) region; 7) survey location; and 8) experience (years). The academic background predictor represented observer’s educational background and included High school and undergraduate (BS and BA), Masters (MS or MA), and PhD. The employment predictor variable represented the vocation of an observer and included academia, state agency, federal agency, and private company/consultant. The identification frequency (hours per month) predictor represented the number of hours (h) spent identifying mussels per month and included 0, 1–4, 5–10, and >11. The identification frequency (days per year) predictor represented the total number of days (d) spent identifying mussels per year and included 0, 1–10, 11–20, >21. The mussel training predictor variable represented where observers learned how to identify mussels and included university, on-the-job training (OJT), university + OJT, and self-taught. The region predictor represented the geographic region of prior mussel experience and included the Midwest, Northeast, Southeast and Southwest, which were defined following US census regions (37). The location variable represented where the survey was administered and included Texas and Ohio. The experience predictor represented total number of years identifying mussels. Other questions relating to personal information about a given observer, such as gender or age, were not included because we were interested in traits that directly or indirectly assess experience with mussel identification. Observers were then assigned to 1 of the 50 stations, each with one of the individual *L*. *teres* whose sex was determined. Observers were then given approximately 1 minute per specimen to provide a sex determination and were not allowed to revisit problem specimens or the example photo.

Based on these questions, the observers who participated were primarily wildlife biologists (98% or 115 observers) with mostly advanced degrees (69% or 81) and having a wide range of experience working with mussels (median = 4 years, range = 0 to 35 years). The observers included state (43% or 50) and federal agency personnel (9% or 10), university students and faculty (32% or 36), and private consultants (16% or 18). The observers received their formal mussel training from a variety of sources, including university (23% or 27), on-the-job training (38% or 45), university plus on the-job-training (17% or 20), and self-taught (21% or 25). The observers varied in where they routinely work with mussels, though all were from the United States, such that 25% (29) identified as working in the Midwest, 9% (10) in the Northeast, 22% (26) in the Southeast, and 44% (52) in the Southwest. Taken together, our observers represented a sample of experienced and inexperienced biologists with mussels from the Northern, Midwest, and Southern United States.

We fitted logistic regression models relating misidentification of sex to personal background information. Following Shea et al. [31], the dependent variable was the observer identification of sex for a given specimen and was coded a 1 for any instance where sex was misidentified and 0 otherwise. Predictor variables were also binary-coded as 0 (background trait absent) or 1 (background trait present), except for observer experience, which was a continuous variable. We suspected that repeated identifications by the same observer may result in autocorrelation among observations, so we included random effects (i.e., individual observer), which represented variation not accounted for by personal background information. We used Bayesian Markov chain Monte Carlo (MCMC) generalized linear mixed models (GLMMs) as implemented in the mcmcGLMM package [37] for R (R Core Team 2017) to develop posterior probability distributions for model parameters. We ran the MCMC chains for 550,000 iterations with a 50,000 iteration burn-in and thinned the posterior sample by a factor of 100, resulting in an effective sample size of 4,500 posterior distribution samples per parameter per chain. Priors were specified following Hadfield [37] for categorical data. To check convergence of the MCMC simulations, we visualized the posterior probability distribution of parameters and interpreted them for fit following guidance provided by Hadfield [37].

We used an information-theoretic approach [38] to evaluate the relative fit of candidate models relating misidentification of sex with observer personal background information. Specifically, we treated the 8 personal background questions as individual hypotheses and evaluated model fit for each one using the Deviance Information Criterion (DIC), which is a Bayesian measure of model fit. DIC weights ($w_{i}$), which range from 0 to 1, were calculated, and the model with the highest weight was considered to be the best-approximating model [38]. We also fitted a random effect only model, to test whether improvement in DIC for a given model was better than not taking into account any of the predictors. We consider models to be plausible if their ΔDIC ≤ 2. For the best-approximating models, odds [39] and median odds ratios (MOR) [40] were calculated to aide in interpretation of parameter estimates on misidentification for fixed and random effects, respectively. We also calculated the 95% highest posterior probability density (95% HPD) intervals for parameter estimates and odds ratios to assess their precision.

### Morphometric analysis

Three morphometric analyses for shell shape were conducted. For traditional morphometrics, ratios of height/length, width/length, and width/height were calculated and normalized using an arcsine-transformation to standardize the variables for size. For geometric morphometrics, we used the software tpsDig v2.10, [41] to place 27 landmarks at the intersection of the shell margin and radial lines extending below the horizontal line. Procrustes transformation was performed using CoordGen6 in the IMP package [42] to remove size from landmark coordinates. For Fourier morphometrics, shell outline was described by 20 Fourier coefficients using Shape v1.3 [43,44].

Morphological variation within and between sexes was analyzed through principal component analysis (PCA), which requires no *a priori* group assignments and simplifies description of variation among individuals. Multivariate analysis of variance (MANOVA) was used to compare between sexes and discriminant function analysis (DFA) was conducted to determine how frequently principal component (PC) scores correctly distinguished between sexes. We used the first 10 PC axes (three PC axes for traditional morphometrics) for MANOVA and DFA. Traditional and geometric morphometric analyses and pairwise comparisons between sexes were performed in the software PAST [45], while Fourier morphometric statistical analysis was done through the SHAPE software [43].

We also surveyed whether shape of males and females at Yegua Creek was characteristic of this species so that broader inferences could be drawn from our results. To do this, we compared morphological variation between populations from Yegua Creek and the East Fork of the Trinity River. All morphometric analyses showed overlapping morphology between populations (S1 Fig). As a result, we concluded that male and female morphotypes of *L*. *teres* from Yegua Creek was typical of the overall sexual dimorphism found within this species. We then included all individuals from both Yegua Creek and the East Fork of the Trinity River to examine morphological variation across sex. We also assessed observer misidentification rates relative to morphological variation but only for the Yegua Creek samples because observers were only surveyed on those specimens.

## Results

### Identification survey

A total of 5,850 identifications were made by 117 observers on 50 specimens of known sex at two academic conferences in 2017. Median observer misidentification rate was ~20% and ranged from 2 to 44% irrespective of the sex of the mussel. Rates were different between sexes as median error rate for males was ~23%, though it ranged from 0 to 63%, whereas for females it was ~10% and ranged from 0 to 50% (Table 1). The best approximating logistic model relating misidentification of sex to observer background information was the mussel training model that included OJT and observer-level random effect. Three other models had ΔDIC values of ≤ 2 and based on DIC weights ($w_{i}$) the mussel training model was 1.4, 2.0 and 2.3 times more plausible than these, which included region (Southwest or Midwest) or location of survey (Texas), plus the observer-level random effect (Table 2). For geographic region of prior experience, median misidentification rates across the Midwest, Northeast, Southeast, and Southwest of the United States were ~22%, 23%, 19%, and 16%, respectively (Table 1). With regards to where observers learned how to identify mussels, median misidentification rates by academia, on-the-job training (OJT), self-taught, and academia + OJT were ~22%, 14%, 21%, and 20%, respectively. Finally, median error rates based on location of where the survey was administered was ~0.16 and 0.21 for the Texas and Ohio locations, respectively (Table 1).

**Table 1:** Summary of misidentification rates by personal background information, sex of specimen, and location of where the survey was administered. N (number of observers), median, min, max, and 25th and 75^th^ percentile summarize the central tendency and spread of misidentification rates per background information trait.

Type	Trait	N	Median	Min	Max	25^th^	75^th^
Academic background	HS/BS	36	0.2	0.06	0.42	0.12	0.24
Academic background	MS/MA	65	0.18	0.04	0.44	0.14	0.26
Academic background	PHD	16	0.2	0.02	0.36	0.14	0.28
Employment	Academia	36	0.22	0.02	0.38	0.16	0.32
Employment	Federal	10	0.16	0.1	0.34	0.13	0.18
Employment	Private	18	0.2	0.08	0.32	0.14	0.22
Employment	State	50	0.19	0.04	0.44	0.13	0.26
Frequency(hours per month)	0	44	0.19	0.04	0.44	0.12	0.27
Frequency(hours per month)	1 to 4	28	0.2	0.02	0.36	0.14	0.25
Frequency(hours per month)	5 to 10	19	0.18	0.1	0.36	0.16	0.24
Frequency(hours per month)	>11	26	0.21	0.08	0.42	0.15	0.27
Frequency(days per year)	0	27	0.18	0.06	0.44	0.12	0.23
Frequency(days per year)	1 to 10	33	0.2	0.04	0.38	0.12	0.28
Frequency(days per year)	11 to 20	16	0.19	0.02	0.36	0.16	0.32
Frequency(days per year)	>21	41	0.2	0.08	0.42	0.14	0.24
Mussel training	Academia	27	0.22	0.06	0.38	0.13	0.31
Mussel training	On-the-job training (OJT)	45	0.14	0.02	0.36	0.12	0.2
Mussel training	Academia + OJT	20	0.21	0.1	0.42	0.16	0.28
Mussel training	Self-taught	25	0.2	0.08	0.44	0.18	0.24
Region	Midwest	29	0.22	0.1	0.42	0.18	0.32
Region	Northeast	10	0.23	0.14	0.38	0.2	0.28
Region	Southeast	26	0.19	0.02	0.36	0.13	0.27
Region	Southwest	52	0.16	0.04	0.44	0.12	0.22
Location	Location of the survey—Ohio	66	0.21	0.02	0.42	0.17	0.28
Location	Location of the survey—Texas	51	0.16	0.04	0.44	0.12	0.22
Sex of specimen	Male	30	0.23	0	0.63	0.17	0.33
Sex of specimen	Female	20	0.1	0	0.5	0.05	0.15
Experience*	Total observer experience	117	4	0	35	1	8

*Summary statistics describe central tendency and spread of total years of experience for observers who participated in the survey and not misidentification rates.

-Note that the total number of participants (N) for a given trait may vary depending on whether or not a response was provided by a given participant for that trait.

**Table 2:** Model type, predictor variables, Deviance Information Criteria (DIC), ΔDIC, and DIC weights ($w_{i}$) for the candidate set of logistic regression models relating misidentification of sex with personal background information. DIC is a measure of model fit, ΔDIC measures the relative difference between the best model (ΔDIC = 0) and all subsequent models in the model set, and $w_{i}$ is the relative likelihood of a model given the data.

Model type	Candidate model	DIC	ΔDIC	$w_{i}$
Academic training	On-the-job training (OJT)	5717.39	0.00	0.28
Region	Southwest	5718.11	0.72	0.20
Location	Location of the survey	5718.80	1.42	0.14
Region	Midwest	5719.10	1.71	0.12
Academic training	Academia	5720.24	2.85	0.07
Academic training	Academia + OJT	5720.30	2.91	0.07
Random effect only	~ + Observer	5720.39	3.00	0.06
Frequency (days per year)	11 to 20	5720.40	3.01	0.06
Academic training	Self-taught	5720.48	3.09	0.06
Employment	Academia	5720.49	3.11	0.06
Region	Southeast	5720.60	3.21	0.06
Region	Northeast	5720.60	3.22	0.06
Employment	Private	5720.69	3.31	0.05
Education	MA	5720.71	3.32	0.05
Frequency (hours per month)	>11	5720.72	3.33	0.05
Education	HS/BS	5721.01	3.62	0.05
Employment	Federal	5721.05	3.67	0.05
Frequency (hours per month)	0	5721.14	3.75	0.04
Education	PhD	5721.22	3.83	0.04
Employment	State	5721.29	3.90	0.04
Fequency (hours per month)	5 to 10	5721.34	3.95	0.04
Frequency (days per year)	1 to 10	5721.34	3.95	0.04
Frequency (days per year)	>21	5721.60	4.21	0.03
Experience (years)	Total observer experience	5721.63	4.24	0.03
Frequency (days per year)	0	5721.69	4.30	0.03
Frequency (hours per month)	1 to 4	5721.88	4.50	0.03

Parameter estimates for models with ΔDIC values of ≤ 2 indicate strong negative relationships between mussel training, region (except for the Midwest model) and location of the survey (Tables 2 and 3). For mussel training, odds-ratios suggest that observers who learned mussel identification as part of their job were ~0.7X less likely to misidentify sex. Odds ratios for region of prior mussel experience indicate that observers from the Southwestern United States were also ~0.7X less likely to falsely identify sex. In contrast, observers whose prior experience was from the Midwestern United States were 1.4X more likely to misidentify sex. Odds ratios also suggest that observers who took the survey in Texas were ~0.7X less likely to falsely identify sex. Finally, observer-level random effects suggested that substantial variability remained among observers’ ability to correctly identify sex after accounting for observer background information. Based on the best-fitting model (mussel training), the MOR for the observer-level random effect suggested that two observers with the same OJT, the less experienced person is 1.5X more likely to misidentify sex. For all parameter estimates, the highest posterior probability density (95% HPD) intervals did not overlap with zero.

**Table 3:** Parameter estimates, standard errors (SE), 95% highest posterior probability density (95% HPD) intervals, odds ratios (OR), and median odd ratios (MOR) based on logistic regression models relating misidentification of sex with personal background information.

Model	Estimate	SE	95% CI		OR/MOR	95% CI	
			Lower	Upper		Lower	Upper
**Mussel training**							
*Fixed effects*							
Intercept	-1.559	0.002	-1.691	-1.417			
OJT	-0.364	0.002	-0.594	-0.131	0.694	0.552	0.877
*Random effects*							
Intercept (observer)	0.193	0.001	0.094	0.289	1.520	1.339	1.670
**Region**							
*Fixed effects*							
Intercept	-1.548	0.002	-1.692	-1.396			
Southwest	-0.339	0.003	-0.564	-0.140	0.712	0.569	0.869
*Random effects*							
Intercept (observer)	0.194	0.001	0.104	0.300	1.522	1.360	1.686
**Location of survey**							
*Fixed effects*							
Intercept	-1.559	0.002	-1.710	-1.415			
Location (Texas)	-0.305	0.003	-0.543	-0.091	0.737	0.581	0.913
*Random effects*							
Intercept (observer)	0.200	0.001	0.111	0.300	1.532	1.374	1.686
**Region**							
*Fixed effects*							
Intercept	-1.773	0.002	-1.892	-1.631			
Midwest	0.303	0.003	0.045	0.548	1.354	1.046	1.730
*Random effects*							
Intercept (observer)	0.202	0.001	0.109	0.300	1.535	1.370	1.686

### Morphometrics

**Fig 1:**
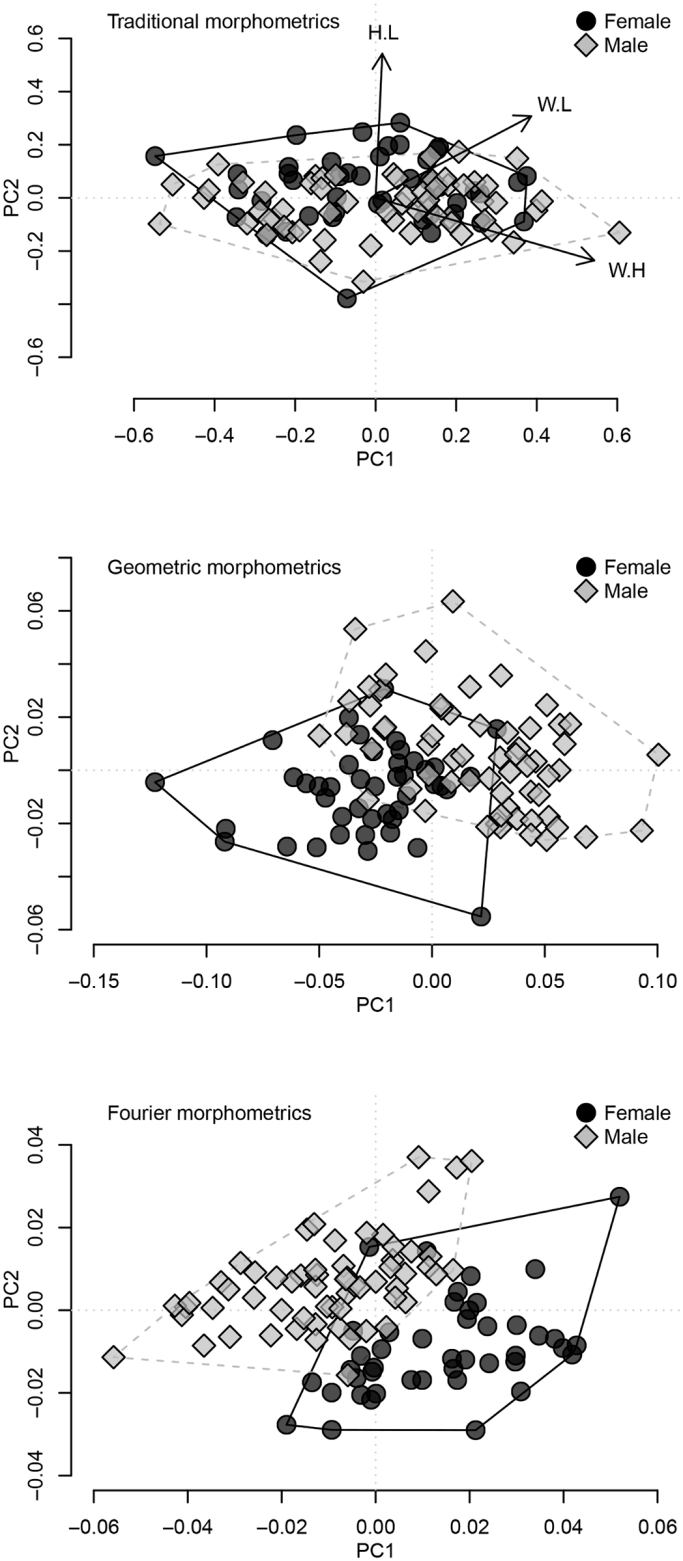
Biplots from principal component analysis (PCA) of traditional morphometrics. (A), geometric morphometrics (B), and Fourier morphometrics (C). Colors and shapes of points correspond to females (black circle; *n* = 44) and males (gray diamond; *n* = 61) of *Lampsilis teres* (Yellow Sandshell) from Yegua Creek and the East Fork of the Trinity River. Polygons enclose convex hulls of each sex (solid line = females; dashed line = males).

**Fig 2:**
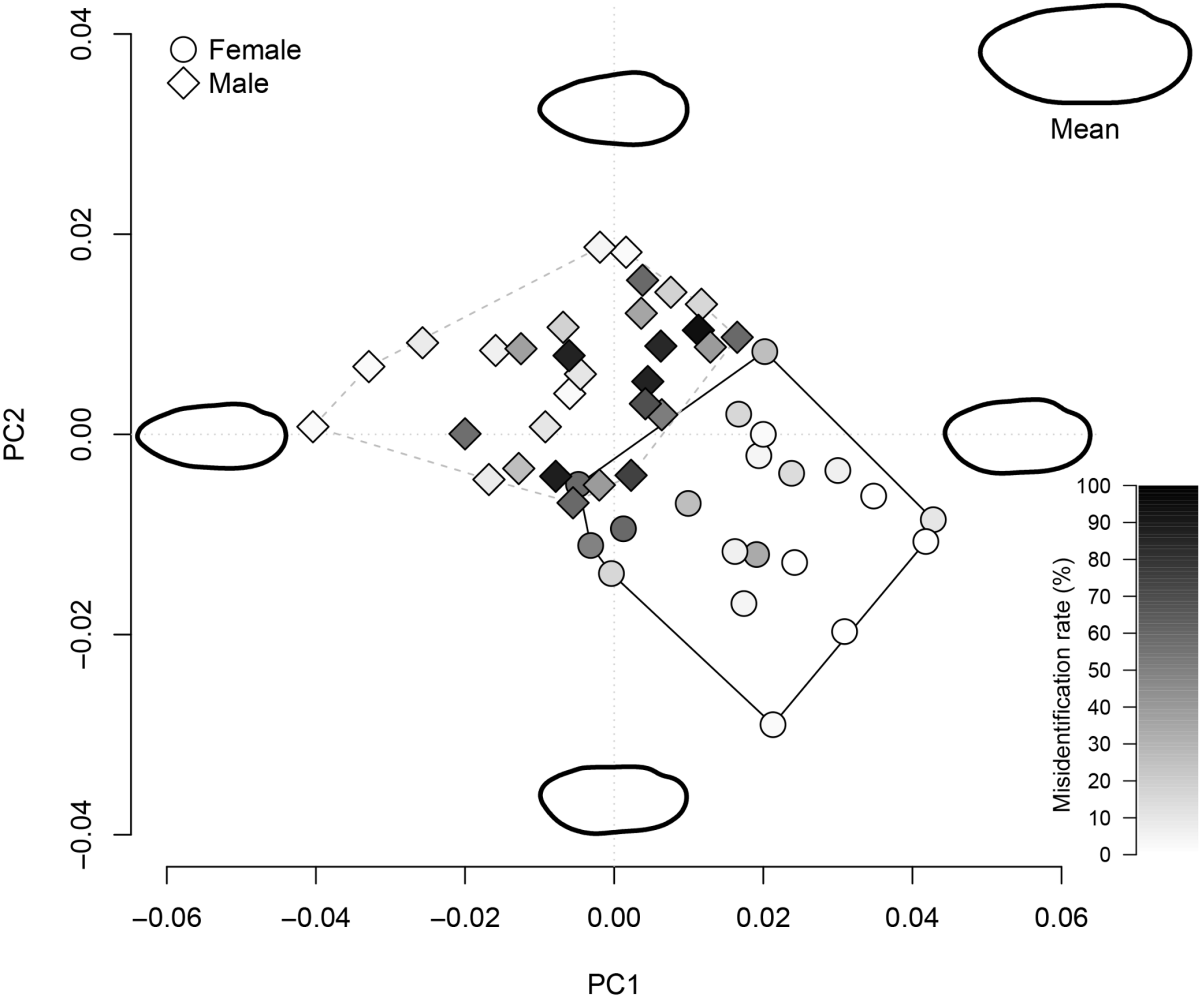
Biplot from principal component analysis (PCA) of Fourier morphometrics. Shapes of points correspond to female (circle; *n* = 20) and males (diamond; *n* = 30) of *Lampsilis teres* (Yellow Sandshell) from Yegua Creek; gradient colors correspond to observer misidentification rates for each specimen. Polygons enclose convex hulls of each sex (solid line = females; dashed line = males). Outlined shell shapes represent a mean shape (top-right) and ± 2 × standard deviations on PC1 and PC2 axes.

We examined 44 females and 61 males of *L*. *teres* from two populations. For traditional morphometric analysis, PCA yielded two distinct eigenvalues and described >99% of the total variability among individuals; the PC1 axis described 80.75% and the PC2 axis described 19.25% of total variation. The PCA plot with groups assigned by sexes showed overlapped morphology (Fig 1), although the centroids were significantly different (Wilk’s Λ = 0.923, *F*_3,10_ = 2.805, *P* = 0.044) and the DFA correctly assigned 60% of individuals to the correct sex. For geometric morphometric analysis, PCA yielded 14 distinct eigenvalues and described >99% of the total variability among individuals; the PC1 axis described 57.0% and the PC2 axis described 13.94% of the total variation (Fig 1). The centroid of morphological variation for males and females were statistically significant (Wilk’s Λ = 0.2022, *F*_10,94_ = 37.08, *P* < 0.001) and the DFA revealed 95.2% of individuals were assigned to the correct sex. For the Fourier analysis, the PCA yielded 26 distinct eigenvalues and described >99% of the total variability among individuals; the PC1 axis described 50.44% and the PC2 axis described 21.3% of the total variation (Fig 1). Morphological differences between sexes were statistically significant (Wilk’s Λ = 0.211, *F*_10,94_ = 35.13, *P* < 0.001) and the DFA correctly assigned 96.2% of individuals to the correct sex. In contrast to traditional morphometrics, the PCA plots from the geometric and Fourier morphometrics showed distinct morphological variation between sexes with little overlap. Overlaying the biplot from the Fourier analysis with misidentification rates for each specimen showed that observers had difficulty in distinguishing males with intermediate morphotypes (Fig 2).

## Discussion

Relatively few studies have considered the prevalence and effect of misidentification of sex in wildlife management and conservation (but see [2–4]) and we are unaware of any studies that have examined this issue and contributing factors for freshwater mussels. We feel this is a problem, because failure to account for errors in sex assignment means there is very little recourse for assessing technique and data limitations that may allow for adjustments during status assessments and recovery planning [2]. For example, we found that observers, some of whom have many years of experience in mussel sex identification, were correct in diagnosing only 80% of the specimens, though there were some who scored much lower, indicating identification rates are greatly varied among observers, regardless of experience. High error rates were largely driven by misidentification of male specimens as observers were on average correct in diagnosing 90% of the female specimens compared to only 77% of the male specimens. Similar results have been observed for other species besides mussels, which suggest this problem may be more widespread [2–4]. In contrast, correct classification of sex for the morphometric approaches exceeded 95%, regardless of sex. Generally, mussels collected during field surveys often have eroded, dirty shells and surveyors are typically limited in the amount of time they have to determine species and sex. This makes correct identification of sex more difficult in the field and so our results likely represent a best-case scenario, meaning field misidentification rates for mussels are likely much higher than what our study showed.

In our study, observers were surveyed on 20 females and 30 males, a sex ratio of 0.7. Under best-case scenario, one where we assume misidentification rates in the field reflect those observed in this study (i.e., 0.10 and 0.23 for females and males, respectively), observers would have reported a sex ratio that could have varied by ± 2 females and 8 males or 0.55 to 1.22, respectively. Although the effect of skewed sex ratios on populations of unionid mussels is not well understood [5], deviation from sex ratios of natural populations causes threats to population viability, particularly in small populations. For example, Wedekind [1] showed using theoretical models that the intrinsic rate of population growth is enhanced if sex ratios are female biased because effective population size (*N*_*e*_) is expected to increase over generations. In contrast, male-biased populations, regardless of the number of generations, reduce *N*_*e*_ over time. Finally, skewed sex ratios can induce Allee effects such as the inability to find mates particularly in small populations [46]. Assuming these predictions apply to mussels, a surveyor could have reached two very different conclusions with the results from the identification survey (i.e., a sex ratio that varies by 0.55 to 1.22): effective population size is expected to increase or it is expected to decrease over subsequent generations. Depending on which outcome is correct, a surveyor could recommend conservation and management actions that are either unnecessary and as a consequence tie up resources that could be used elsewhere or are destructive as researchers would fail to recognize that population viability was declining. This example underscores the potential negative management and conservation implications of biased sex data on demographic assessments of viability.

We found that misidentification rates were unrelated to profession and experience per se, but were associated with geographic region of prior mussel experience and where observers learned how to identify mussels. This result contradicts conventional wisdom that more experience equates to lower identification error, which has been documented for mussel species identification [31] and detection during field surveys [47]. Regarding geographic region of experience, observers that were from the Southwestern United States had lower misidentification rates compared to those from other regions of the United States. This finding is noteworthy because most of the observers that identified as being from this region were from Texas, which also explains why observers who took the survey in Texas were less prone to error than those from other regions. Over the last 5 years, state and federal agencies within Texas have invested heavily in mussel related research, training, and outreach in response to the potential listing of 12 mussel species under the U.S. Endangered Species Act [48–51]. This would seem to indicate that training could help with reducing misidentification of sex, though overall misidentification rates for the Southwestern United States was still high (median error rate of 19%). So, it is uncertain if additional training can reduce misidentification of sex to a level that results in minimal bias to estimated sex ratios. In contrast, we found that observers from the Midwestern United States were more prone to misdiagnosing sex, which is difficult to explain as identification training occurs regularly within this region. Within parts of the Midwest, *L*. *teres* is a species of conservation concern, but is not federally protected and so it may be rarely encountered during field surveys, which may explain our results. Another potential explanation is that mussel training within this region primarily focuses on species identification not sex identification, which is typical of most mussel training. Finally, differences between regions could also be related to subtle regional changes in allometry, sexual dimorphism, and geographic variability in shell morphology. Although we surveyed for this between two distant sites in Texas, we are unable to say whether inter-regional variability exists for *L*. *teres* and if so how that affects identification of sex. We also found that observers who learned mussel identification as part of their job were better able to determine sex than someone who was solely trained in an academic setting. In the United States, mussel identification training is typically offered through state and regional workshops, though identification training does occur at some universities but tends to be species or project specific. Thus, we interpret this result as another line of evidence, albeit indirect, that workshops can help with improving misidentification of sex. Shea et al. [31]came to a similar conclusion, but with respect to species misidentifications. The authors of that study recommended regional workshops as one way to improve species misidentification rates, which we support, but add that at those workshops experts needs to be teaching sex identification skills for species that are sexually dimorphic, which should include both laboratory and field-based exercises. Reference specimens in these workshops should encompass known morphological variation and their sex previously determined using gamete sampling (e.g., [26,52]) or histology (e.g., [14,27–30]).

The lack of association between factors related to experience and misidentification demonstrates that identification of sex based only on shell morphology may be an unreliable method. However, it could also be related to how we phrased our questions. For instance, instead of asking how many years or hours per month observers spent identifying mussels, we could have asked how much time individuals spent diagnosing sex based solely off morphology. Rephrasing the question may have helped explain why surveyor experience, by month or year, failed to explain misidentification rates. Similarly, rephrasing the questions regarding employment and mussel training to include specific references to sex identification based on morphology may have accounted for the lack of association between these factors and misidentification rates. We suspect that rephrasing the questions would not have changed the results of this study because: 1) a majority of the observers surveyed were state, regional, and national experts in various aspects of mussel conservation and so they were familiar with sexual dimorphism in mussels; and 2) observers, on average, with little to no experience did as well as those with many years of experience.

Hermaphrodism and protandry are other potential explanations for identification error of sex. In general, mussels are dioecious but there are a small number of species that can become hermaphroditic or even change sex under different environmental conditions [5]. *Lampsilis teres* to the best of our knowledge is not known to demonstrate either trait. However, if *L*. *teres* were hermaphroditic, the syringe technique, the method used in this study, may not have detected both male and female gametes because they likely would have been separated spatially within the gonadal tissue. Finally, the degree and extent to which mussels are hermaphroditic or change sex is not well understood [5] and so it remains unclear whether this is an issue that natural resource managers and researchers should be concerned with when determining or using sex ratio data.

**Fig 3:**
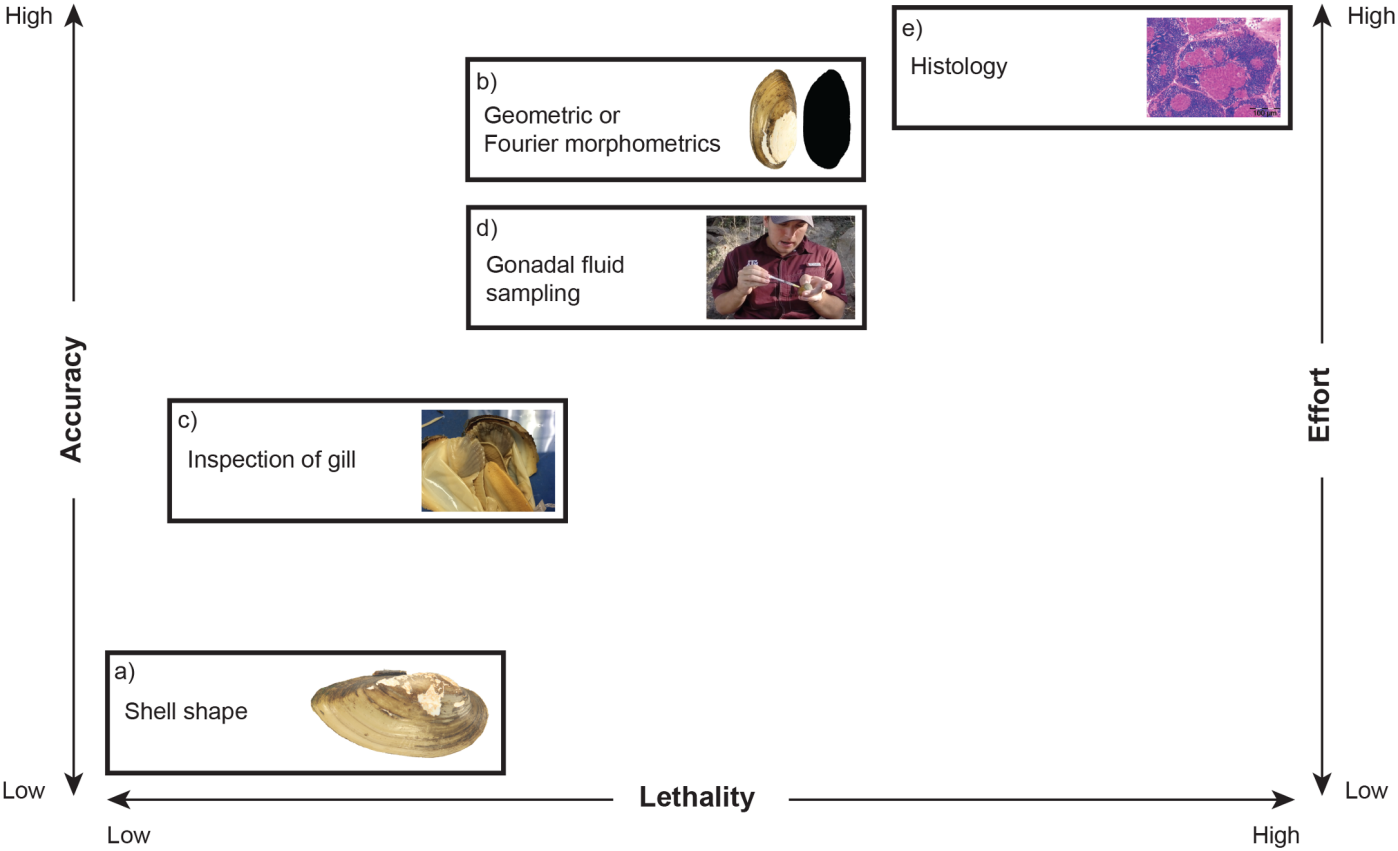
Flow chart exploring pros and cons of different approaches to determine sex for mussels as it relates to time, effort, lethality, and accuracy. Note that Geometric or Fourier morphometrics have similar accuracy as gonadal fluid sampling but requires more effort.

Despite little available guidance on methods for identifying sex of mussels, we used our results and the literature to formulate a flow chart exploring pros and cons of different approaches as it relates to effort, lethality, and accuracy of a particular method (Fig 3; S1 Table). It is important to point out that although our recommendations are specific to mussels, we feel this approach could serve as a guide for other taxa where misidentification of sex is suspected to be an issue. That said, in our proposed schema identification of sex using shell morphology is the easiest method for assigning sex, in terms of effort, and is non-lethal, but is also inaccurate as we have shown with this study (Fig 3a). This approach is frequently used [18,19,53–55] but should be abandoned until it can be demonstrated that error rates are less than what was shown in this study or if it is used only to provide preliminary estimates of sex that are then surveyed using more robust methods. Shell analysis with the aid of morphometric and shape recognition approaches could provide more reliable estimates of sex based on external morphology and has been used with other aquatic taxa [56–58] (Fig 3b). However, this requires a computer program that can characterize morphology of a given specimen and compare it to a reference library of validated external morphologies (i.e., using the morphometric approach demonstrated in this study with histology or gonadal fluid sampling—see below for pros and cons of both methods) for each sex. Thus, the morphometric approach presented in this paper could serve as an example for how to do this for other sexually dimorphic mussel species. During reproductive seasons, sex can be determined by inspecting the gills to determine if an individual is gravid [20–23,59–61] (Fig 3c). This method is frequently used and more correct than determinations based solely on external morphology. However, it is more costly in terms of effort, can cause reproductive failure as gravid mussels could abort their brood, and may result in mortality if inappropriately done. It is also not useful for separating non-gravid females from males. In addition to diagnosing gravidity, anatomy of the mantle such as the presence of caruncles or a mantle lure can also be used to help distinguish females vs. males, but these characteristics can be difficult to identify in the field, particularly for the inexperienced researcher. Lastly, gonadal fluid sampling (Fig 3d) and histology of reproductive structure (Fig 3e) are the most precise methods for determining sex of mussels but are also the costliest in terms of effort. The two methods differ in that gonadal fluid sampling is taken from live individuals and thus does not require vouchering like histology, though mortality can occur from gonadal fluid sampling, especially if not done properly, but histology will be more correct if the species in question is hermaphroditic.

Our study indicates that using external morphology to diagnose sex is prone to high error rates. Our results in combination with those of Shea et al. [31] suggest that misidentification, whether it be species or sex, is prevalent among unionid researchers, even those regarded as experts. The use of identification workshops may help with reducing error associated with identifying male vs. female based on external morphology, but until this is demonstrated we recommend researchers take gonadal fluid or voucher individuals for histology if sex ratios are to be analyzed. Given that misidentification of species and sex have the potential to bias genetic and demographic estimates and assessments of population viability, we strongly urge that state and federal agencies require researchers, whether it be academics or biologists from state agencies or private industry, to demonstrate proficiency prior to being issued a collection/research permit. In states like Ohio and Virginia this is common practice [62,63] and has had a measurable effect on reducing species misidentification rates, though specific instruction on sex identification should be included. We suspect similar results if this were extended to diagnosing sex using external morphology. Finally, because identification of sex for most wildlife species typically relies on external morphology [2–4], we think our results could be indicative of a much larger and perhaps more systemic issue that includes other species outside of mussels. Thus, efforts should be made to determine error rates in estimation of sex for other taxa, particularly for species of high conservation concern where misidentification of sex ratios could lead to inappropriate management strategies that at best waste precious resources and at worst hasten their decline.

## Supporting information

S1 Fig Biplots from principal component analysis (PCA) of traditional morphometrics (A), geometric morphometrics (B), and Fourier morphometrics (C) comparing morphological variation of *Lampsilis teres*, Yellow Sandshell, between populations from Yegua Creek and the East Fork of the Trinity River. Colors and shapes of points correspond to Yegua Creek (black circle; *n* = 50) and East Fork of the Trinity River (gray diamond; *n* = 61).
(TIF)

S1 Table Summary of pros and cons of methods used to diagnose sex of mussels. (DOCX)
