# Supplementary figures and images for: Misidentification of sex for Lampsilis teres, Yellow Sandshell, and its implications for mussel conservation and wildlife management
Source: PLoS One. 2018 May 16;13(5):e0197107. doi: 10.1371/journal.pone.0197107 (PMC5955573; doi:10.1371/journal.pone.0197107)

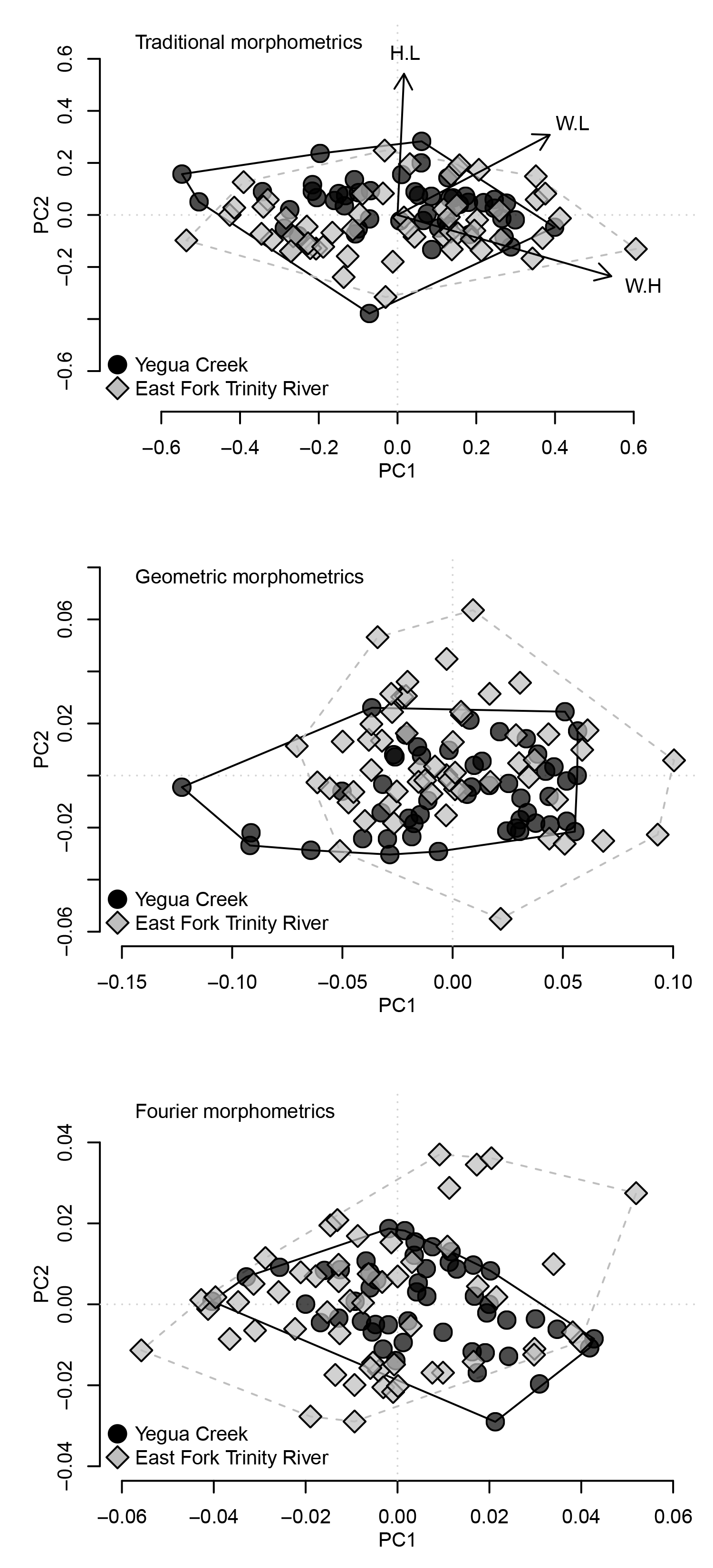

Supplement: S1 Fig — Colors and shapes of points correspond to Yegua Creek (black circle; n = 50) and East Fork of the Trinity River (gray diamond; n = 61). (TIF) [file pone.0197107.s001.tif]
