# Supplementary material for: Misidentification of sex for Lampsilis teres, Yellow Sandshell, and its implications for mussel conservation and wildlife management
Source: PLoS One. 2018 May 16;13(5):e0197107. doi: 10.1371/journal.pone.0197107 (PMC5955573; doi:10.1371/journal.pone.0197107)
Supplement: S1 Table — (DOCX) [file pone.0197107.s002.docx]

S1 Table

| **Method** | **Pros** | **Cons** | **Reference** |
| --- | --- | --- | --- |
| Shell shape | - Can be done in the field - non-lethal - Can be used with fossil records or extinct species - Inexpensive - Not time-consuming | - Some species are not sexually dimorphic; - Shell shape influenced by environmental conditions, parasitism, and age - Misidentification common | Kirtland (1834); Heard and Guckert (1970); Kotrla and James, 1987); Berger et al. (2001); Zieritz and Aldridge (2011) |
| Shape analysis | - Can be done in the field - non-lethal - Can be used with fossil records or extinct species - Not time-consuming - Misidentification uncommon | - Technology is available for fish but not mussels so currently unavailable - Expensive | Zieritz and Aldridge (2009); Minton and Wang (2011); Lakshmanan (2012); Benitez (2013); Joaquinto et al. (2017) |
| Inspection of gills | - Can be done in the field - non-lethal - Moderately accurate - Inexpensive - Not time-consuming | - Difficult to separate non-gravid females and males - Variability in timing and duration of brooding - Evidence of brooding is difficult to distinguish for some species - Misidentification can be common | Campbell (1969); Barnhart and Baird (2000); Rogers et al. (2001); Jolley et al. (2004); Agatsuma et al. (2005); Williams and Babcock (2005); Petes et al. (2008) |
| Gonadal fluid sampling | - Can be partially done in the field - non-lethal - Misidentification uncommon | - Long-term effects on growth and reproduction unknown - Moderately expensive - Time-consuming | Bauer (1987); Baird (2000); Christian et al. (2000); Henley (2002); Shiver (2002); Haag and Station (2003); Moles and Layzer (2008); Saha and Layzer (2008); Galbraith and Vaughn (2009); Tsakiris et al. (2016) |
| Histological | - Misidentification uncommon | - Lethal - Cannot be done in the field - Expensive - Time-consuming | van der Schalie and van der Schalie (1963); van der Schalie (1970); Downing et al. (1989); Jirka and Neves (1992); Alfaro et al. (2001); Haggerty et al. (2005); Hines et al. (2007); Petes et al. (2008); Haggerty et al. (2011); Fraser et al. (2016) |
